# 

**Published:** 1908-04-04

**Authors:** 


### CONTENTS.

| | PAGE |
|---|---|
| **The Nature of "Idiopathic" Epilepsy** ... ... ... | 1 |
| **The Pathological Aspect of Primogeniture** ... ... | 2 |
| **Annotations—** | |
| Flies, Dust, and Mosquitoes — The Disposal of the Dead—Anæsthetics in America ... ... ... ... ... ... ... | 3 |
| **Medical Opinion and Movement** ... ... ... ... ... | 4 |
| **Hospital Clinics -** | |
| Carcinoma of the Uterus. The Importance of Early Diagnosis: Symptoms and Treatment. By Frederick Bowreman Jessett, F.R.C.S. ... ... ... ... ... ... ... ... | 7 |
| **Special Article—** | |
| The Uses of Hydrogen Peroxide ... ... ... ... ... | 9 |
| **Medicine—** | |
| A Case for Diagnosis ... ... ... ... ... ... ... | 11 |
| **Points in Treatment—** | |
| Bier's Method and Ulcers of the Leg ... ... ... ... | 12 |
| **Points in Surgery—** | |
| Aneurysm ... ... ... ... ... ... ... ... ... | 13 |
| Ankylosis ... ... ... ... ... ... ... ... ... | 14 |
| **Mental Diseases—** | |
| The Proposed London Hospital for the Insane ... ... ... | 15 |
| **Public Health and Hygiene—** | |
| Rubella and its Relations to Scarlet Fever and Measles ... ... | 16 |
| **Pathology—** | |
| The Tissue Changes in Diphtheria ... ... ... ... ... | 17 |
| **Progress in Otology—** | |
| The Operative Surgery of Labyrinthitis ... ... ... ... | 19 |
| **Resident Medical Officers' Department—** | |
| The R.M.O. and the County Court—III. ... ... ... ... | 20 |
| **Hospital Administration—** | |
| Fiji: its Hospitals and Diseases ... ... ... ... ... | 21 |
| News and Coming Events ... ... ... ... ... ... | 23 |
| Social and Poor law Problems ... ... ... ... ... | 24 |
| **Nursing Administration—** | |
| The Uniform Certificate—I. ... ... ... ... ... ... | 25 |
| **The Graduates' Medical Schools** ... ... ... ... | 26 |
| **Editor's Letter-Box—** | |
| Man's Superiority ... ... ... ... ... ... ... ... | 26 |
| **New Appliances and Things Medical—** | |
| A New Clinical Thermometer ... ... ... ... ... ... | 26 |

